# Homeostatic conflict as a driver for brain aging

**DOI:** 10.3389/fgene.2026.1881856

**Published:** 2026-08-28

**Authors:** Cristian A. Wulkop-Gil, Michael Petrascheck

**Affiliations:** 1 Department of Molecular and Cellular Biology, La Jolla, CA, United States; 2 Department of Neuroscience, The Scripps Research Institute, La Jolla, CA, United States

**Keywords:** aging, cell type imbalance, dementia, ferroptosis, homeostatic conflict, neurodegeneration, senescence, therapeutic strategies

## Abstract

Mammalian tissue function depends on the balance between proliferative, mitotic cell populations and irreplaceable post-mitotic cells. Throughout life, aging progressively disrupts cell type balance and thus tissue function through opposing forces on different cell types. Aging promotes the aberrant proliferation or entry into pro-inflammatory senescent states of mitotic cells, while contributing to the functional erosion and, in vulnerable populations, loss of post-mitotic irreplaceable cells. The divergent age-associated vulnerabilities of mitotic and post-mitotic cells caused by aging generate a case of *homeostatic conflict*, in which tissue maintenance simultaneously demands the elimination of damaged mitotic cells and the preservation of irreplaceable ones. We examine the concept of *homeostatic conflict* using the aging brain as an example, and illustrate how the senescence-prone glia expand and acquire SASP-rich transcriptional states, while post-mitotic neurons undergo widespread loss of molecular identity and cell death, resulting in cell type imbalance and consequently tissue dysfunction. Based on the observed *homeostatic conflict,* we then devise potential context-sensitive therapeutic strategies by combining interventions that protect from ferroptosis to selectively protect irreplaceable cells with interventions that eliminate maladaptive ones to resolve the age-associated cell type imbalance and thus the underlying *homeostatic conflict*. We present *homeostatic conflict* as a concept that goes beyond the reductionist view in which numerous isolated processes promote aging in isolation, and provides a logical framework that integrates multiple processes and explains how their interaction drives age-associated dysfunction in a manner instructive to the development of pharmacological combination strategies.

## 1 Aging and homeostatic conflict

Antagonistic pleiotropy, proposed by George Williams, is one of the leading evolutionary explanations for why aging persists and is not selected against (Williams, 1957; Li et al., 2023). Its central idea is that the activity of individual genes is pleiotropic and not specific to single traits. While its effect on one trait may be beneficial, its effect on another can be simultaneously detrimental. As natural selection acts most strongly before or during the reproductive period, genes that boost fertility but promote aging are retained in the population, as their advantage early in life outweighs their negative effect later. The opposing actions of the same gene on different traits highlight an evolutionary conflict, in which the optimal level of gene activity is set by a balance between its opposing effects on different traits.

Such pleiotropic and opposing effects of gene activities are not confined to evolutionary or developmental timescales, as they can drive opposing effects on different physiological systems within an individual. As an illustration of this, a high-protein diet helps older individuals to maintain muscle mass while straining renal function, generating a conflict that must be balanced to maximize benefit and minimize harm (Bauer et al., 2013; Ko et al., 2020). Analogously, inflammatory eicosanoid signaling protects against infection, but, when sustained as chronic low-grade inflammation, commonly referred to as inflammaging (Franceschi et al., 2000), it contributes to cardiovascular disease or dementia (Ridker et al., 2017; Heneka et al., 2015). We use the term *homeostatic conflict* to describe situations like this, in which a single age-associated process is simultaneously adaptive for one system or cell population and maladaptive for another (Table 1). Consequently, a therapeutic intervention intended to correct one maladaptive process may antagonize a coexisting adaptive one, offsetting any net benefit. *Homeostatic conflict* is conceptually related to antagonistic pleiotropy, but rather than explaining the evolution of aging, it provides a framework for evaluating therapeutic intervention related to aging.

**TABLE 1:** Homeostatic conflict across biological processes and interventions.

Function	Process / intervention	Adaptive role	Maladaptive consequence
Redox & iron	Iron	O₂ transport, mitochondrial respiration, myelination	Fenton chemistry, ferroptosis
	Ferroptosis inhibition	Neuronal survival	Potential tumor growth
	Reactive oxygen species	Redox signaling, adaptation (hormesis)	Lipid peroxidation, ferroptosis
	NRF2 antioxidant response	Cytoprotection, redox defense	Tumor survival, chemoresistance
Senescence	Cellular senescence	Wound healing, tumor suppression	SASP-driven inflammation
	Senolytic clearance	Removes SASP-secreting cells	Ablates reparative senescent cells
	p53 activity	Tumor suppression	Senescence, stem-cell exhaustion
Proteostasis & growth	Protein synthesis	Regeneration, repair	Protein aggregation
	Autophagy	Clears damaged organelles and aggregates	Excessive self-digestion, cell death
	mTOR signaling	Growth, protein synthesis, anabolism	Drives senescence, accelerates aging
	GLP-1 receptor agonism	Weight and fat loss	Muscle loss
Neuroimmune	Glutamate signaling	Excitatory neurotransmission, plasticity	Excitotoxicity, neuronal death
	Complement / microglial pruning	Synapse refinement, debris clearance	Excessive synapse loss, neurodegeneration
	Inflammation	Host defense, immunity	Chronic inflammaging, CVD, dementia

Representative processes, resources, and interventions for which the same activity is simultaneously adaptive in one context and maladaptive in another. Each entry lists an adaptive role (green) and a maladaptive consequence (red); the opposition between them defines the *homeostatic conflict* that constrains age-related therapeutic strategy. Entries are grouped by biological system (left).

An example of *homeostatic conflict* arises from the different demands of various cell types necessary for tissue function. Tissue homeostasis depends on the precise balance between proliferating, mitotic cell populations and post-mitotic cells that have permanently exited the cell cycle (Biteau et al., 2010; Ruijtenberg and van den Heuvel, 2016). This balance is not static but shifts continuously with age (López-Otín et al., 2023). As organisms age, mitotic cells accumulate damage that can drive aberrant proliferation or entry into a pro-inflammatory senescent state, while post-mitotic cells suffer gradual functional erosion or, if vulnerable, are lost outright (López-Otín et al., 2023; Campisi, 2013). Here, we outline the concept of *homeostatic conflict* between cell types in the brain, describe how *homeostatic conflict* is exacerbated by aging, impairing tissue function, and its importance to the development of aging therapeutics.

## 2 Homeostatic conflict resulting from cell type imbalance in the aging brain

The brain offers a compelling illustration of how age disrupts cell type balance by opposing forces. The organ contains both proliferating glial cells (e.g., astrocytes, microglia, and oligodendrocytes) and post-mitotic neurons that are both irreplaceable and indispensable to sustained cognitive and motor function. Adult neural stem cells (NSCs) are a third cell population that has the capacity to generate new neurons (Obernier and Alvarez-Buylla, 2019). However, this regenerative output is minor, restricted to a few regions of the brain, and declines sharply with age (Kalamakis et al., 2019; Dulken et al., 2019; Sorrells et al., 2018). The coexistence of proliferating senescence-prone glia and the loss of post-mitotic irreplaceable neurons, within a single organ, exemplifies a *homeostatic conflict* that requires the coordinated activation of cell-death and cell-survival pathways throughout the tissue to preserve cell type balance and tissue function.

The molecular signatures of this tissue-balance interplay in the aging brain have now been recorded at single-cell resolution by several studies and synthesized in recent reviews (Jin et al., 2025; Jeffries et al., 2025; Sun et al., 2025). A comprehensive brain-wide single-cell transcriptomic atlas of the aging mouse brain–which profiled over 1.2 million cells across 16 regions in 2- and 18-month-old mice–found that mature neurons *largely* survive into late middle age in terms of cell number, but already undergo widespread downregulation of synaptic, axonal, and dendritic gene expression programs (Jin et al., 2025). This bulk survival in late middle age, however, conceals the already ongoing selective vulnerability of specific circuit-critical neuronal populations. In humans, *postmortem* stereological analyses in the entorhinal cortex confirm a neuron loss of approximately 2 million neurons from 11.7 million neurons in middle-aged adults to 9.7 million in individuals over 70, independent of Alzheimer’s disease-associated tau or amyloid pathology status (Wegiel et al., 2017). Hippocampal CA1 pyramidal neurons, subiculum neurons, and dopaminergic neurons of the substantia nigra follow a similar trajectory (Simic et al., 1997; Fearnley and Lees, 1991; Rudow et al., 2008). Thus, a progressive loss in specific irreplaceable neuron populations is a feature of normal aging that disrupts cell type balance in the aging brain, which likely predisposes older adults to cognitive decline and dementia.

**FIGURE 1:**
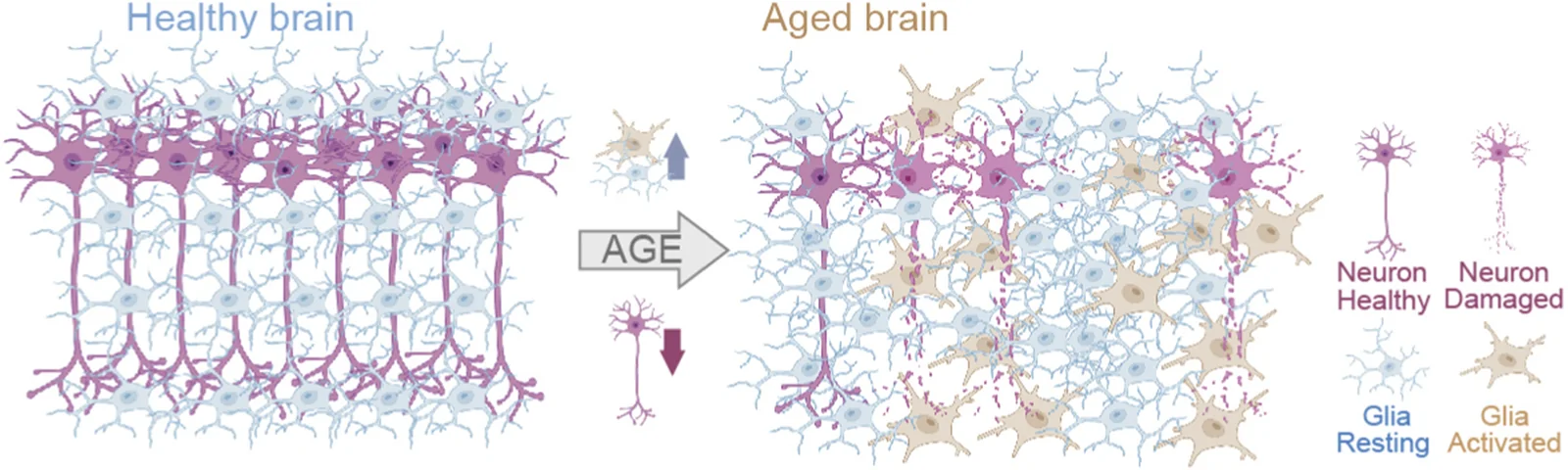
Homeostatic conflict in the Aging Brain: The expansion of senescence-prone glia combined with the loss of neurons results in cell type imbalance and consequently dysfunction generating a case of *homeostatic conflict,* in which tissue maintenance simultaneously demands the elimination of damaged mitotic cells and the preservation of irreplaceable ones. Created in BioRender. Wulkop-Gil, C. A. (2026) https://BioRender.com/amw70x9.

In parallel, proliferating glial cells follow a fundamentally different course, representing the mitotic arm of the aging cell population imbalance (Allen et al., 2023; Hammond et al., 2019; Wong, 2013). Rather than gradually losing identity, aging microglia expand in number, particularly in subcortical regions (Grabert et al., 2016). This expansion is itself heterogeneous. Microglial turnover estimates range from largely long-lived, slowly renewing populations to more rapidly renewing pools depending on region and species (Füger et al., 2017; Réu et al., 2017). Therefore, glial expansion is best understood as a dominant tendency rather than a strict cell-cycle dichotomy. Astrocytes, meanwhile, undergo region-dependent morphological remodeling and altered territorial organization (Grosche et al., 2013; Popov et al., 2021), and select populations of both cell types acquire aberrant, pro-inflammatory, and senescent states (Sala Frigerio et al., 2019; Liddelow et al., 2017; Bhat et al., 2012; Cohen and Torres, 2019; Iram et al., 2016). The brain thus ages through a bifurcated process. Neurons quietly lose molecular identity and decline in number, while glia proliferate and shift toward inflammatory, senescent-like states that amplify damage (Figure 1). In this example, aging generates a *homeostatic conflict* by generating a progressive imbalance favoring expanding, senescence-prone glia at the cost of non-dividing neurons. Its resolution requires the simultaneous promotion of cell removal (inflammatory and senescent-like glial cells) and cell survival (neurons), to restore balance. In these next sections, we will discuss the therapeutic challenges created by *homeostatic conflict* and how its resolution by simultaneously protecting neurons from ferroptosis and reducing maladaptive glia with senolytics provides therapeutic opportunities.

## 3 Ferroptosis and the aging brain

Recent evidence has implicated ferroptosis in neuronal loss in age-associated dementia (Stockwell, 2022; Hambright et al., 2017). Ferroptosis was formally defined as a distinct form of regulated cell death in 2012 by Dixon and colleagues, who identified it while characterizing the mechanism of action of erastin (Dixon et al., 2012). This small molecule selectively kills RAS-mutant cancer cells in a manner morphologically, biochemically, and genetically distinct from apoptosis and necrosis. The name was coined to reflect its absolute dependence on iron, and its biochemical core is the iron-catalyzed peroxidation of polyunsaturated fatty acid-containing phospholipids (PUFA-PLs) in cellular membranes. Ferroptosis is held in check primarily by the glutathione peroxidase 4 (GPX4)–glutathione (GSH) axis, as well as iron and PUFA-PL availability (Yang et al., 2014). Cells undergo ferroptotic cell death when this axis is overwhelmed through depletion of GSH, direct inhibition of GPX4, or accumulation of labile iron driving Fenton chemistry and runaway lipid peroxidation. Notably, a mechanistically related or potentially identical form of oxidative cell death, then termed oxytosis, had been described in neurons 2 decades earlier, induced by glutamate-mediated depletion of cystine import through system Xc^−^ and subsequent GSH collapse (Murphy et al., 1989; Lewerenz et al., 2018). Additional suppressor pathways, including the FSP1–CoQ10 axis, provide further layers of ferroptosis resistance whose expression varies across cell types, adding a critical dimension of context-dependence to the pathway’s regulation (Doll et al., 2019; Bersuker et al., 2019; Mao et al., 2021; Kraft et al., 2020). Our interest, however, is not confined to this lethal endpoint. The same iron-dependent chemistry also operates sublethally, remodeling redox balance, membrane composition, and transcriptional state in cells (Dar et al., 2026; Coradduzza et al., 2023). We therefore treat ferroptosis as the most sharply defined endpoint of a broader peroxidative biology whose dysregulatory effects, on surviving neurons and glia alike, may matter as much for the aging brain as the death it names. Throughout, we accordingly distinguish three terms. *Iron dyshomeostasis*, the dysregulated iron handling that expands the labile, redox-active pool; *lipid peroxidation*, the resulting oxidative modification of membrane PUFA-PLs; and *ferroptosis*, iron-dependent lipid peroxidation-mediated regulated cell death. The first two are upstream events rather than synonyms for ferroptotic death, and their by-products accumulate across many forms of cellular stress.

The aging brain provides particularly fertile ground for Fenton chemistry (Fei and Ding, 2024; Wang et al., 2024). Iron accumulates progressively with age through increased transferrin receptor expression, dysregulated ferritin storage, and impaired export (Lu et al., 2017; Ashraf et al., 2018). The iron accumulation expands the pool of redox-active labile iron available to catalyze lipid peroxidation (Levi et al., 2024; Mezzanotte and Stanga, 2025; Long et al., 2023). Simultaneously, the antioxidant defense erodes. GSH availability declines through reduced cystine import via system xc^−^, decreased GSH synthesis, and increased oxidation to GSSG (Currais and Maher, 2013). Concomitantly, GPX4 expression declines in the aged brain, lowering the theoretical threshold for ferroptotic execution (Jamerson et al., 2026).

Consistent with these converging vulnerabilities, markers of lipid peroxidation and antioxidant failure, including elevated lipid peroxidation products (4-HNE, MDA), depleted GSH, and reduced GPX4 levels, have been identified in *postmortem* brain tissue from Alzheimer’s disease, Parkinson’s disease, and ALS patients (Bellinger et al., 2011; Chen et al., 2015; Butterfield and Halliwell, 2019). Age-associated iron accumulation is particularly pronounced in the substantia nigra, hippocampus, and entorhinal cortex, regions discussed above as sites of measurable neuronal loss and progressive age-related functional decline (Lu et al., 2017; Ashraf et al., 2018). The substantia nigra shows severe disruption of iron storage and export precisely in the dopaminergic subregion most vulnerable to age-related neuronal loss (Ashraf et al., 2018; Fearnley and Lees, 1991; Walker et al., 2016). Together, these observations show that the lipid peroxidation–iron axis is spatially and temporally associated with the region-specific neuronal loss that characterizes normal aging and neurodegeneration (Song et al., 2026; Wang et al., 2022; Pandey and Rizvi, 2015), suggesting the involvement of ferroptosis.

Beyond outright death, sublethal ferroptotic stress–lipid peroxidation below the threshold for ferroptotic execution–may also contribute to the age-related erosion of neuronal functional identity (Maher et al., 2025). Lipid peroxidation products, particularly 4-HNE, form adducts with transcription factors, proteasomal components, and DNA, which disrupt gene expression programs that maintain neuronal identity (Li et al., 2022). The transcriptional erosion of neuronal identity documented in aging brains may thus partly reflect a response to chronic, sublethal peroxidative stress that precedes, and perhaps precipitates, ferroptotic execution in the most vulnerable neurons.

This stress is not confined to neurons. Microglia are major iron-buffering cells whose storage capacity saturates with age (McCarthy et al., 2018; Adeniyi et al., 2023). The resulting sublethal peroxidative stress may push microglia and iron-dysregulated astrocytes toward pro-inflammatory, senescence-like states (Russo et al., 2025; Liddell et al., 2024). Lipid peroxidation and iron dyshomeostasis may therefore act upstream of the paracrine damage inflicted on neurons, positioning sublethal peroxidative stress as one driver of SASP-competent glial states (Coradduzza et al., 2023; Masaldan et al., 2019).

Taken together, ferroptosis represents one age-associated force that directly and indirectly drives the loss of functional neurons, contributing to a progressive cell type imbalance and *homeostatic conflict* through the depletion of cells.

## 4 Cellular senescence in the aging brain

The peroxidative stress described above converges on a second axis of glial dysfunction. Senescence is a stress-induced, essentially irreversible cell-cycle arrest accompanied by persistent DNA-damage signaling, resistance to apoptotic death, and acquisition of a senescence-associated secretory phenotype (SASP) rich in pro-inflammatory cytokines and proteases (Rodier and Campisi, 2011; Rodier et al., 2009; Coppé et al., 2008; Yosef et al., 2016). In the aging brain, senescence is predominantly observed in glia. Systematic *in vitro* profiling of human brain cell types finds astrocytes and oligodendrocyte-lineage cells among the most senescence-prone and SASP-active, whereas neurons are comparatively resistant to replication-associated senescence induction and express fewer canonical SASP hallmarks (Russo and Riessland, 2026). Nevertheless, neurons are not wholly exempt and can enter senescence-like states under stress (Russo et al., 2025; Herdy et al., 2022).

The pathological weight of glial senescence lies in its non-cell-autonomous reach. Senescent astrocytes secrete SASP factors, including IL-6, IL-8, and CCL5 (Bhat et al., 2012). Conditioned media from senescent astrocytes activate microglia and induce neuronal death (Zhang et al., 2022). Moreover, senescent astrocytes downregulate the glutamate transporters EAAT1 and EAAT2, producing excitotoxic stress and neuronal death in co-culture (Limbad et al., 2020). In subcortical regions, senescent glial populations expand and acquire a persistent SASP (Grabert et al., 2016) that propagates senescence to neighboring cells through paracrine signaling (Russo and Riessland, 2026), promoting a feed-forward cycle of neuroinflammation that accelerates brain-wide aging (Gulen et al., 2023).

Taken together, senescence and the expanding populations of activated glial cells represent a second age-associated force contributing to a progressive cell type imbalance and *homeostatic conflict* through the generation of maladaptive cells.

## 5 Homeostatic conflict as a therapeutic challenge

As outlined in the two previous sections, ferroptosis and senescence represent two age-associated forces that drive tissue imbalance from two directions. They simultaneously deplete functional neurons while generating maladaptive glial populations (Figure 1), setting up a *homeostatic conflict* whose therapeutic resolution requires the simultaneous preservation and depletion of distinct cell populations.

Preclinical evidence for ferroptosis inhibition as a neuroprotective strategy in age-related neurodegeneration is substantial and growing (Chen et al., 2021; Mahoney-Sánchez et al., 2021). Canonical ferroptosis inhibitors, ferrostatin-1 (Fer-1) and liproxstatin-1 (Lip-1), have been tested in cellular and mouse models of Alzheimer’s disease. In PSEN1-mutant iPSC-derived organoids, Fer-1 prevents Aβ aggregation, reduces lipid peroxidation, and restores iron storage (Majerníková et al., 2024). In APP/PS1 mice, Fer-1 and Lip-1 improved memory and reduced lipid peroxidation and oxidative stress markers (Bao et al., 2021). Fer-1 also protects dopaminergic neurons from α-synuclein-induced death *in vitro* (Majerníková et al., 2025), consistent with *postmortem* findings that GPX4 localizes to surviving nigral neurons and dystrophic striatal axons in Parkinson’s brain, where total nigral GPX4 is reduced alongside broader evidence of iron dysregulation and ferroptosis vulnerability (Bellinger et al., 2011; Levi et al., 2024).

The first clinical attempts to therapeutically exploit these insights, however, have been sobering. Iron chelation with deferiprone, a brain-penetrant chelator, showed encouraging neuroprotection in animal models of Alzheimer’s, Parkinson’s, and Huntington’s disease and clinical benefit in neurodegeneration with brain iron accumulation (Prasanthi et al., 2012; Carboni et al., 2017; Agrawal et al., 2018; Abbruzzese et al., 2011). Yet, randomized trials in both early Parkinson’s disease and amyloid-confirmed early Alzheimer’s disease found that deferiprone paradoxically accelerated clinical decline (Ayton et al., 2025; Devos et al., 2022). The failure of this trial is particularly instructive as deferiprone met its target-engagement endpoint, lowering brain iron as intended. The most parsimonious interpretation is that the effect of iron on pathology and health is context dependent. Chelation does not distinguish between the redox-active labile pool that drives ferroptotic peroxidation in vulnerable neurons from the tightly buffered iron required for dopamine synthesis, mitochondrial respiration, and myelin maintenance across the same tissue (Levi et al., 2024). This result highlights a case of *homeostatic conflict* where iron is both poison and antidote, dependent on its molecular context, and where unidirectional manipulation ameliorates one physiological function at the expense of another. Iron-directed neuroprotection will therefore likely require a context-dependent therapeutic strategy that is cell-type- or state-selective rather than a global depletion.

Senolytic compounds selectively eliminate senescent cells by exploiting their dependence on anti-apoptotic survival pathways (He and Sharpless, 2017; Zhu et al., 2015). In PS19 mice, genetic ablation of p16-positive cells, or the senolytic BCL-2/BCL-xL inhibitor navitoclax reduced tau phosphorylation and neurofibrillary tangles, attenuated gliosis, preserved neurons, and prevented cortical atrophy (Bussian et al., 2018). Clinical evidence is preliminary but encouraging (Hickson et al., 2019; Gonzales et al., 2022; Gonzales et al., 2023). A 12-week pilot in older adults at risk for Alzheimer’s disease, using pulsed D + Q, was well-tolerated and modestly improved cognitive scores in participants with the lowest baseline performance, with TNF-α reductions tracking cognitive improvement consistent with SASP suppression (Millar et al., 2025). Larger Phase 2 trials are underway (Orr, 2025). Together, these establish proof-of-concept that reducing the SASP-secreting glial burden can attenuate cell-non-autonomous damage to neurons (Bussian et al., 2018; Ogrodnik et al., 2021; Millar et al., 2025).

The senolytic strategy rests on the assumption that neuronal damage is predominantly driven by paracrine signals from senescent neighbors and is therefore largely reversible upon their removal (Baker and Petersen, 2018; Lee et al., 2021; da Silva et al., 2019). Yet the preclinical record is inconsistent. D + Q reduced tau pathology in rTg4510 mice (Musi et al., 2018) but failed to lower senescence markers or improve cognition in P301S tau mice despite prolonged treatment (Riordan et al., 2023), and in aged rTg4510 mice both D + Q left cognition unchanged while worsening frailty (Garbarino et al., 2025).

As seen for iron, and consistent with the existence of another *homeostatic conflict* that arises with age, senescence is also not uniformly pathological. Transient senescent states contribute to wound healing, tissue remodeling, limitation of fibrosis, and tumor suppression (Muñoz-Espín and Serrano, 2014), and distinct senescent populations can exert directly opposing effects within a single tissue, such that ablating one protects while ablating another aggravates injury (Zhao et al., 2024). In the CNS, senescence markers overlap substantially with reactive, activated, and stress-responsive states that are not necessarily deleterious. Context-insensitive senolysis risks removing cells still performing protective functions (Russo et al., 2025; Muñoz-Espín and Serrano, 2014). Recognizing this, the field has moved toward “precision senolytics” that selectively eliminate pathogenic subsets through surface-antigen-restricted CAR-T cells or β-galactosidase-activated prodrugs, thus sparing beneficial cells (Amor et al., 2020; Barthet and Lowe, 2025). Such selectivity is especially pertinent in the brain, where senescence localizes to distinct glial, endothelial, and neuronal senotypes that are challenging to distinguish by a single senolytic (Russo et al., 2025), and whose net effect in the aged, comorbid brain remains untested. Taken together, broad senolysis alone is neither reliably effective nor free of harm, motivating its pairing with a neuroprotective arm rather than its use as a standalone strategy.

## 6 Resolution of homeostatic conflict as a therapeutic opportunity

The therapeutic challenges described in the previous section share *homeostatic conflict* as a common denominator. While the concept of *homeostatic conflict* is intuitive, the notion that aging exacerbates *homeostatic conflicts* and its relevance to therapeutic development is rarely considered. *Homeostatic conflict* immediately implies a therapeutic combination approach to address the problem. To our knowledge, nearly all therapeutic frameworks consider pathways and drug targets in isolation. We currently lack strategies to devise combination therapies for age-related diseases whose logic is directly rooted in the biology of aging. The framework of *homeostatic conflict* attempts to fill this gap.

Considering cognitive decline as a result of a *homeostatic conflict* generated by the age-associated rise in cell type imbalance suggests a two-directional combination strategy that pairs a non-chelating ferroptosis inhibitor to protect neurons with a senolytic to clear glia (Pandey, 2025). Such a combination would confront two genuinely independent but interacting problems: The protection of vulnerable post-mitotic neurons from lipid peroxidation (Chen et al., 2021; He and Sharpless, 2017) and the excessive senescent-cell burden that drives pathology independently through SASP-driven damage.

The main opportunity lies in the assumption that the two sides of a *homeostatic conflict* are likely to reinforce each other, resulting in a negative synergism that stabilizes a pathological equilibrium. Signaling by inflammatory glia may increase the susceptibility of neurons to ferroptosis, whose death releases oxidized lipids, further promoting the inflammatory state of glia (Coradduzza et al., 2023). The resolution of such a conflict by a bidirectional combination approach is thus likely to result in a therapeutic synergism resolving pathology.

The challenges in resolving a *homeostatic conflict*, however, are practical rather than fundamental. The senolytic arm must be selective. Broad senolysis risks removing context-dependent reparative senescent cells, and CNS senescence markers overlap with reactive, stress-adaptive states that are not uniformly pathogenic (Muñoz-Espín and Serrano, 2014; Neumann et al., 2023). Delivering two agents to an aged, comorbid population also compounds pharmacological risk, where polypharmacy, altered drug clearance, and cumulative off-target toxicity are already substantial concerns (Davies et al., 2020). This constraint necessitates either highly selective agents or a single rationally designed polypharmacological molecule engaging both targets (Avchaciov et al., 2025).

Encouragingly, broad anti-ferroptotic protection of vulnerable neurons should not meaningfully shield senescent glia from apoptotic clearance (Zhu et al., 2015). Neuronal protection and selective senolysis could thus operate in parallel, each addressing the compartment that the other cannot. Even more encouragingly, multiple plant polyphenols have already been identified that protect from ferroptosis and reduce senescence in the aging or injured brain, though for most, the two activities have been demonstrated in different settings. Baicalein, a 12/15-lipoxygenase inhibitor, blocks ferroptosis, reduces neuronal death *in vivo* (Li et al., 2019), and suppresses astrocytic senescence and SASP output via JAK2/STAT1/NF-κB *in vitro* (Gao et al., 2021). Apigenin blocks myeloperoxidase-mediated ferroptosis in the injured brain *in vivo* (Shao et al., 2020). It acts as a mechanistically defined senomorphic through the PRDX6–iPLA2 axis in non-neural cells (Zhang et al., 2025), with senescence markers reduced *in vitro* in aged astrocytes (Cavalier et al., 2024). Honokiol protects neurons from ferroptosis via SIRT3-mediated GPX4 regulation *in vivo* (Zeng et al., 2025) and reduces senescence markers in microglia *in vitro* (Sasia et al., 2024). While these plant molecules are senomorphs that suppress SASP, Fisetin combines ferroptosis inhibition with genuine senolytic activity, selectively killing senescent cells (El Ashmawy et al., 2025; Yousefzadeh et al., 2018). It thus provides a proof-of-concept for a state-selective, single-agent intervention with the potential to resolve a central *homeostatic conflict* in the aging brain. A phase 2 trial in older adults with mild cognitive impairment and Alzheimer’s disease is ongoing.

Here we have used the brain to illustrate the concept of *homeostatic conflict* and how aging-associated cell-type imbalance may contribute to cognitive impairment. We illustrated the challenges posed by *homeostatic conflict* for therapeutic development using the example of iron chelation and how classical unidirectional reductionist approaches run the risk of replacing one pathology for another. We then showed how the framework of *homeostatic conflict* can guide therapeutic design, proposing that ferroptosis inhibition paired with senolysis could resolve, or at least stabilize, the cell-type imbalance in the aging brain. The specific strategy we present is hypothetical, but the framework is not. *Homeostatic conflict* grounds combination therapy in the biology of aging, moving beyond *ad hoc* pairings assembled without regard for how interventions interact in an aging system. As it is highly unlikely that aging and its diseases will be conquered by a single drug modulating one specific pathway, strategic frameworks like *homeostatic conflict* that are rooted in the biology of aging are necessary for the development of future combination therapeutics, as the enormous number of possible combinations makes empirical testing impossible (López-Otín et al., 2023).
